# Patient satisfaction with out-of-hours primary care in the Netherlands

**DOI:** 10.1186/1472-6963-5-6

**Published:** 2005-01-15

**Authors:** CJT van Uden, AJHA Ament, SO Hobma, PJ Zwietering, HFJM Crebolder

**Affiliations:** 1 Department of Integrated Care, Research Institute Caphri, University Hospital Maastricht, P.O. Box 5800, 6202 AZ Maastricht, the Netherlands; 2 Department of General Practice, Research Institute Caphri, Maastricht University, P.O. Box 616, 6200 MD Maastricht, the Netherlands; 3 Department of Health Organization Policy and Economics, Research Institute Caphri, Maastricht University, P.O. Box 616, 6200 MD Maastricht, the Netherlands

## Abstract

**Background:**

In recent years out-of-hours primary care in the Netherlands has changed from practice-based to large-scale cooperatives. The purpose of this study is to determine patient satisfaction with current out-of-hours care organised in general practitioner (GP) cooperatives, and gain insight in factors associated with this satisfaction.

**Methods:**

From March to June 2003, 2805 questionnaires were sent to patients within three weeks after they had contacted the GP cooperative in their region. The study was conducted in the province of Limburg in the South of the Netherlands. One-third of these questionnaires was sent to patients who had only received telephone advice, one-third to patients who attended the GP cooperative for consultation, and one-third to patients who received a home visit. Four weeks after the first reminder, a non-respondents telephone interview was performed among a random sample of 100 patients. Analyses were performed with respect to the type of consultation.

**Results:**

The total response was 42.4% (1160/2733). Sixty-seven percent of patients who received telephone advice only reported to be satisfied with out-of-hours care. About 80% of patients who went to the GP cooperative for consultation or those receiving a home visit, reported to be satisfied. Factors that were strongly associated with overall satisfaction included, the doctor's assistant's attitude on the phone, opinion on GP's treatment, and waiting time.

**Conclusion:**

Patients seem generally satisfied with out-of-hours primary care as organised in GP cooperatives. However, patients who received telephone advice only are less satisfied compared to those who attended the GP cooperative or those who received a home visit.

## Background

In recent years, out-of-hours primary care in the Netherlands has been substantially reorganised. Formerly, general practitioners (GPs) used to perform these services in small locum groups (6 to 8 GPs) in which they joined a rota system. Nowadays, out-of-hours care is organised in large-scaled GP cooperatives (45 to 120 GPs) following examples in the UK and Denmark [1,2].

The initiative of reorganising out-of-hours care has come mainly from the profession itself, motivated by increased dissatisfaction with the organisation of former out-of-hours primary care services. This dissatisfaction was mainly due to the high perceived workload (after out-of-hours service a regular day of work followed), and poor separation between work and private life. The main advantage of the reorganisation was the substantial reduction of number of hours a GP has to be on call. Furthermore, the organisation of out-of-hours care became much more professional by installing management, employing doctor's assistants, and using chauffeured cars. Studies have indicated that GPs appear to be generally satisfied with out-of-hours care organised in cooperatives[3].

Not only did things change for doctors, but also patients experienced some important changes in out-of-hours primary care. Generally, the reorganisation caused a shift from more personal care to more anonymous care, with increased distance to the GP. Formerly, when patients needed primary care outside office hours, the probability of being seen by their own or a local GP with whom they were familiar, was higher. In addition, when patients contacted the GP during out-of-hours in the past, they were most likely to speak to the GP himself on the phone. Nowadays, the phone is staffed by a doctor's assistant who decides what action should follow the patient's call. Moreover, out-of-hours care used to be delivered by local GPs, indicating short distances to the GP's practice. In large-scale GP cooperatives, the distance to a GP outside office hours will have increased substantially for most patients.

We expected that patient satisfaction would have been reduced after the reorganisation, because factors that guaranteed personal out-of-hours care at a short distance, that may be important to patients, were changed substantially. Furthermore, in Denmark it has been shown that after the out-of-hours primary care reform patient satisfaction dropped significantly[4,5].

Patient satisfaction with out-of-hours primary care has quite often been investigated, especially in the UK [4-11]. Mostly, comparisons have been made between different types of out-of-hours services. Several of these studies focused on out-of-hours primary care as organised in GP cooperatives. These studies have shown that patients are generally satisfied with out-of-hours primary care organised in GP cooperatives[5,8,9,11]. Nevertheless, patients receiving telephone advice only, appear to be less satisfied compared to those attending the cooperative or those receiving a home visit. In addition, it has been shown that the patient's expectation about their contact with the GP cooperative strongly affects the patient's overall satisfaction with out-of-hours care[12]. Other variables that appear to be related to overall satisfaction are, access to a car, age, and waiting time[8].

Insight in patient satisfaction with out-of-hours care supplies the health care provider with important information on the patient's perception of the quality of that care. During the last years, Dutch GP cooperatives have often received negative publicity in newspapers. The reorganisation has had some important implications for patients, and therefore research on their opinions about current out-of-hours care is warranted. The purpose of this study is to determine patient satisfaction with current out-of-hours care, and to determine how satisfaction is related to different aspects of the patient's contact with a GP cooperative.

## Methods

### Setting

The study was conducted in the province of Limburg in the South of the Netherlands. With respect to out-of-hours primary care, the province is organisationally divided in five regions. Two of these regions each have two GP cooperatives (NL and ML), one region (OZL) has one GP cooperative with two satellite centres, and in the other two regions (WM and MH) only one GP cooperative is operational. All cooperatives but one (MH) are organisationally separate from the emergency department of the local hospital, and are located nearby the hospital. This implies that patients may choose between attending the emergency department and the GP cooperative for medical problems during out-of-hours. The MH cooperative is located at the emergency department of the region's only hospital and sees all patients needing out-of-hours care, except for those having a referral for emergency care.

In total, these seven GP cooperatives cover a population of about 1.1 million people (the total Dutch population is over 16 million people), and are fully operational since the 1^st ^of September 2001.

### Development of the questionnaire

To determine relevant issues for the questionnaire we interviewed GPs and managers involved with out-of-hours primary care. In addition, we analysed the process for a patient contacting the GP cooperative for all three loci of care (telephone advice, consultation at the cooperative, and home visits) separately to make sure that all facets of the GP cooperative a patient faces would be incorporated in the questionnaire. Moreover, we also analysed unpublished Dutch questionnaires in this field, and the patient satisfaction questionnaire developed by McKinley et al. [13]. Based on these three analyses, we identified a number of relevant elements (initial scales). Next a set of items was developed to enable us to produce multi-item scales. Subsequently, this list was sent to the patient organisation in our province, the two largest health insurance funds, and to the five GP cooperative organisations for commentary. These organisations were asked to critically review the list of items, and to add or remove items if they considered it necessary. After receiving all commentary the questionnaire was adjusted and was submitted to five people not involved in the development but with experience with out-of-hours primary care to check for clarity of the questions.

Finally three questionnaires were constructed for each of the three types of consultations (telephone advice, consultation at the cooperative, and home visit). The three questionnaires differed on items related to the specific type of contact, but general items were the same for all three questionnaires. In this way it was possible to avoid complex skip sections which lengthen the questionnaire and can reduce the response rate. We used a balanced Likert five point scale (strongly agree, agree, neutral, disagree, strongly disagree) to record responses.

The questionnaire related to telephone advice contained six initial scales measuring: accessibility of the cooperative by phone, doctor's assistant's attitude, questions asked by the assistant, advice given by the assistant, urgency of patient's complaint, and overall satisfaction.

The questionnaire related to consultations at the cooperative contained ten initial scales: accessibility of the cooperative by phone, doctor's assistant's attitude, questions asked by the assistant, urgency of patient's complaint, waiting time at the cooperative, waiting room, distance to the cooperative, GP's attitude, treatment by GP, and overall satisfaction.

The questionnaire related to home visits contained eight initial scales: accessibility of the cooperative by phone, doctor's assistant's attitude, questions asked by the assistant, urgency of patient's complaint, waiting time until GP arrives, GP's attitude, treatment by GP, and overall satisfaction.

In addition, patient characteristics such as, age, gender, level of education, and health insurance (as a measure of social economic status) were recorded. Patients were also asked which type of consultation they expected prior to their contact with the GP cooperative, and whether they thought that the right diagnosis had been made.

### Sample

From March to June 2003 a sample of 2805 patients – who had contacted the GP cooperative in their region – received a questionnaire by mail. Patients received this questionnaire within three weeks after they had contacted the GP cooperative. Sampling was performed per GP cooperative within the four-month period. With respect to patients who received telephone advice only and those who attended the GP cooperative, a computer program randomly selected each fourth patient contact with the GP cooperative backwards from the moment of sampling. Since the number of home visits is limited, all 150 patients, who were visited by a GP from the cooperative, prior to the moment of sampling received a questionnaire. These procedures assured that the time between receiving the questionnaire and the contact with the GP cooperative was not more than three weeks.

Per region 450 questionnaires were sent out; 150 to patients who received only telephone advice, 150 to patients who visited the GP cooperative, and 150 to patients who received a home visit. Because of parallel research, more questionnaires were sent out in one of the regions (WM): 1005 questionnaires equally distributed among the three types of patient contact with the GP cooperative. The study size was chosen based on previous research by McKinley et al[7,13], who presented a study sample of about 1400 patients. We estimated that about half of all questionnaires would be returned, and therefore distributed 2805 questionnaires.

The study was approved by the Institutional Medical Ethics Board.

### Reminder and non-respondents interview

Three to four weeks after the questionnaire had been distributed, a reminder was sent to patients who had not returned the questionnaire, with the exception of the WM area. Four weeks after the last reminder, a random sample of 100 patients who had not responded, was contacted by phone. They were asked about their reasons not to return the questionnaire, and about their opinion on the contact they had with the GP cooperative. This interview was performed during office hours, during a three-week period.

### Statistics

Principal components analysis with varimax rotation was used to test whether the items could be assumed to measure similar aspects or components of patients' opinions about their contact with the GP cooperative. Next, Cronbach's alpha coefficient was calculated to estimate the internal consistency as a measure for reliability for each component. Finally, scale scores were calculated per component by summing the scores per item and expressing the total result as a percentage of the maximum score for each scale[13,14]. Scale scores could range between 0 and 100.

The relationship between individual variables and overall satisfaction was analysed using multiple regression analysis, with subscale satisfaction scores as covariates. Variables that did not significantly contribute to the regression model were excluded from the final model. In case of missing data, listwise deletion of missing cases was applied. All data were analysed using SPSS-pc, version 10.0.5.

## Results

### Patient characteristics

Seventy-two of the 2805 questionnaires were excluded, either because they could not be delivered (patient had moved or gave a wrong address), the patient had died, or the patient was sent a double questionnaire (multiple contacts). Eventually the response was 42.4% (1160/2733). Generally more women responded to the questionnaire, and about three-quarter of the respondents had public health insurance (table 1). The age of respondents of those who received telephone advice only was comparable with those who attended the GP cooperative for a consultation. The respondents who received a home visit were generally older; two-third was over sixty years of age.

**Table 1:** Patient characteristics.

	Telephone advice	Consultation at the GP cooperative	Home visit
	n (%)	n (%)	n (%)

Response	366/908 (40.3)	392/912 (43.0)	402/903 (44.5)

Age			
0 – 20 years	127 (35.5)	146 (39.0)	9 (2.3)
21 – 40 years	96 (26.8)	81 (21.7)	26 (6.6)
41 – 60 years	67 (18.7)	82 (21.9)	93 (23.8)
> 60 years	68 (19.0)	65 (17.4)	263 (67.3)

Total	358 (100)	374 (100)	391 (100)

Gender			
Male	148 (42.3)	159 (48.5)	177 (46.0)
Female	202 (57.7)	169 (51.5)	208 (54.0)

*Total*	*350 (100)*	*328 (100)*	*385 (100)*

Level of education			
Low	92 (27.2)	91 (25.0)	161 (46.4)
Middle	164 (48.5)	188 (51.6)	131 (37.8)
High	82 (24.3)	85 (23.4)	55 (15.8)

Total	338 (100)	364 (100)	347 (100)

Health insurance			

Public	268 (74.4)	283 (73.5)	314 (80.5)
Private	92 (25.6)	102 (26.5)	76 (19.5)
*Total*	*360 (100)*	*385 (100)*	*390 (100)*

### Telephone advice

Forty percent (366/908) of the patients who had received telephone advice only, returned the questionnaire. 67% of these patients responded to be satisfied (44.3%) or very satisfied (22.3%) with their contact with the GP cooperative, and 57% thought that the current out-of-hours care was an improvement compared to the former situation. We identified the same six scales that were initially set to represent patients' opinions on aspects of primary out-of-hours care (table 2). All six scales had Cronbach's alpha coefficients between 0.64 and 0.93. Detailed information on the scales and items can be found in table 7.

**Table 2:** Description of scales representing patients' opinion on different aspects of out-of-hours primary care.

	Cases	Cronbach's alpha	Scale score
Scales ^a^	n		Mean ± SD (95%CI)

**Telephone advice**			

Accessibility by phone	364	0.72	76.5 ± 18.9 (74.6–78.5)
Doctor's assistant's attitude	363	0.91	72.8 ± 22.1 (70.5–75.1)
Questions asked by assistant	361	0.64	58.6 ± 25.4 (56.0–61.3)
Advice given by assistant	351	0.93	53.7 ± 27.3 (50.8–56.5)
Urgency of complaint	363	0.86	69.1 ± 24.5 (66.6–71.7)
Overall satisfaction	361	0.93	64.2 ± 26.1 (61.5–66.9)

**Consultation at the GP cooperative**			

Accessibility by phone	385	0.73	79.3 ± 17.6 (77.5–81.1)
Doctor's assistant's attitude	386	0.88	79.8 ± 16.3 (78.2–81.4)
Questions asked by assistant	384	0.65	63.5 ± 23.0 (61.2–65.8)
Urgency of complaint	384	0.79	72.0 ± 21.5 (69.8–74.1)
Waiting time at cooperative	387	0.62	61.5 ± 25.8 (58.9–64.1)
Waiting room	381	0.60	65.6 ± 20.3 (63.5–67.6)
Distance to cooperative	388	0.75	66.7 ± 21.2 (64.5–68.8)
Treatment by GP	377	0.93	81.0 ± 18.9 (79.1–82.9)
Overall satisfaction	392	0.88	73.7 ± 19.8 (71.7–75.6)

**Home visit**			

Accessibility by phone	391	0.86	80.9 ± 18.4 (79.1–82.7)
Doctor's assistant's attitude	393	0.90	80.6 ± 18.6 (78.7–82.4)
Questions asked by assistant	383	0.73	59.2 ± 26.6 (56.5–61.9)
Urgency of complaint	383	0.78	86.7 ± 16.0 (85.1–88.3)
Treatment by GP	380	0.96	84.4 ± 19.7 (82.4–86.4)
Waiting time until GP arrives	369	-	60.0 ± 30.7 (56.8–63.1)
Overall satisfaction	390	0.92	74.6 ± 22.4 (72.4–76.9)

^a ^Scale scores range from 0 to 100, where 0 represents very dissatisfied and 100 represents highly satisfied.

**Table 7:** Patient satisfaction questionnaire. (Original items are in Dutch)

**Scale 1. Accessibility by phone^t,c,v^**	
It was easy to find the phone number of the GP cooperative^#^	(+)
It was easy to get through on the telephone	(+)
The time until the doctor's assistant picked up the phone was short	(+)

**Scale 2. Doctor's assistant's attitude^t,c,v^**	

The doctor's assistant was friendly on the phone	(+)
The doctor's assistant had enough time to talk to me on the phone	(+)
The doctor's assistant seemed to understand the problem	(+)
The doctor's assistant took my problem seriously	(+)
The information given by the doctor's assistant was very clear	(+)

**Scale 3. Questions asked by the doctor's assistant^t,c,v^**	

The doctor's assistant asked too many questions	(-)
I thought it was annoying that the doctor's assistant started with noting my personal data before asking about my complaints	(-)

**Scale 4. Urgency of complaint^t,c,v^**	

I believed my problem was very severe	(+)
I thought my problem needed immediate care	(+)

**Scale 5. Advice given by doctor's assistant^t^**	

The doctor's assistant's information about my problem was good	(+)
The advice the doctor's assistant gave me was very useful	(+)
The telephone advice by the doctor's assistant had reassured me	(+)
The telephone advice by the doctor's assistant was sufficient considering my problem	(+)
I thought the doctor's assistant was right to give me telephone advice only	(+)

**Scale 6. Waiting time at the cooperative^c^**	

I thought I had to wait too long at the registration desk	(-)
I thought I had to wait too long before the GP came to see me	(-)

**Scale 7. Waiting room^c^**	

There was enough material (magazines et cetera) in the waiting room to entertain the patients	(+)
The waiting room looked very clean	(+)

**Scale 8. Distance to the GP cooperative^c^**	

I think the travel time from my house to the GP cooperative is too long	(-)
The GP cooperative is easy accessible	(+)

**Scale 9. Treatment by the GP^c,v^**	

The GP took my problem seriously	(+)
The GP was friendly	(+)
The GP gave me clear information about my problem	(+)
The advice the GP gave me was very useful	(+)
The GP had enough time for me during the consultation	(+)
I was very pleased with the treatment by the GP	(+)

**Scale 10. Waiting time until GP arrives^v^**	

I thought it took too long for the GP to arrive	(-)

**Scale 11. Overall satisfaction^t,c,v^**	

I am satisfied about this contact with the GP cooperative	(+)
I am satisfied about the time it took to help me	(+)
I think the GP cooperative functions very well	(+)
*Satisfaction rating on a scale from 1 to 10 regarding the functioning of the GP cooperative^‡^*	
Satisfaction rating on a scale from 1 to 10 regarding the telephone procedure at the GP cooperative^‡^,*	

^t ^scale for the patients group who received telephone advice only

^c ^scale for the patients group who attended the GP cooperative for a consultation

^v ^scale for the patients group who received a home visit

* this item was excluded from the scale related to patients who attended the GP cooperative

^# ^this item was excluded from the scale related to patients who received a home visit

^‡ ^these items have been divided by two to reach the same range as the other items.

Overall satisfaction in this group was significantly related to five scales, with a variance explained of 62% (see table 3.). When patients judged that the right diagnosis had been made overall satisfaction was higher. We found that satisfaction also increased with age. When patients were satisfied with the accessibility of the cooperative by phone, the doctor's assistant's attitude on the phone, and the doctor's assistant's advice overall satisfaction was higher.

**Table 3:** Regression analysis with overall satisfaction with out-of-hours primary care as dependent variable of patients who received only telephone advice (adjusted R^2 ^= 0.615).

	Unstandardised coefficients		Standardised coefficients		
	
	B	SE	Beta	t	Sig.
Constant	-2.404	4.302		-0.559	
Diagnosis ^(1 = right, 0 = wrong)^	12.345	2.644	0.200	4.668	< 0.001
Patient's age	0.077	0.036	0.076	2.128	0.034
Accessibility by phone ^a^	0.155	0.054	0.112	2.859	0.005
Doctor's assistant's attitude ^a^	0.401	0.067	0.355	5.960	< 0.001
Doctor's assistant's advice ^a^	0.267	0.055	0.282	4.840	< 0.001

Variables that did not significantly contribute to the regression model: Patient's gender, type of health insurance, level of education, expectation about type of consultation, patient's perceived urgency of his or her complaint, and opinion on the questions asked by the doctor's assistant.

^a ^Scale score ranges from 0 to 100, where 0 represents very dissatisfied and 100 represents highly satisfied.

### Consultation at the GP cooperative

Forty-three percent (392/912) of the patients who attended the GP cooperative returned the questionnaire. Approximately 80% of these patients reported to be satisfied (54.6%) or very satisfied (26.3%) with their contact with the GP cooperative, and 61% thought that the current out-of-hours care was an improvement compared to the former situation. We identified nine scales that represent patients' opinions on aspects of primary out-of-hours care (table 2), with Cronbach's alpha coefficients between 0.62 and 0.93. Two initial scales have been merged into one scale; these were patient's opinion on the GP's attitude and the treatment by the GP. All other identified scales were the same as the initial scales. Detailed information on the scales and items can be found in table 7.

Seven variables proved to be predictors of overall satisfaction, with a variance explained of 51% (see table 4.). Patients, who expected prior to their contact with the cooperative that they were going to be asked to come to the GP cooperative, were generally more satisfied. Those who believed that their medical problem was urgent were less satisfied. Long waiting times and dissatisfaction with the distance to the cooperative also reduced overall satisfaction. When patients were satisfied with the accessibility of the cooperative by phone, the doctor's assistant's attitude on the phone, and the GP's treatment overall satisfaction was higher.

**Table 4:** Regression analysis with overall satisfaction with out-of-hours primary care as dependent variable of patients who went for consultation to the GP cooperative. (adjusted R^2 ^= 0.501).

	Unstandardised coefficients		Standardised coefficients		
	
	B	SE	Beta	t	Sig.
(Constant)	-5.249	5.187		-1.012	
Expectation about contact *	4.313	2.113	0.078	2.042	0.042
Accessibility by phone ^a^	0.095	0.047	0.088	2.022	0.044
Doctor's assistant's attitude ^a^	0.165	0.055	0.138	2.981	0.003
Urgency own complaint ^b^	-0.072	0.036	-0.078	-2.008	0.045
Waiting time ^a^	0.181	0.030	0.241	6.059	< 0.001
Distance to cooperative ^a^	0.176	0.035	0.192	4.965	< 0.001
GP's treatment ^a^	0.454	0.042	0.441	10.756	< 0.001

Variables that did not significantly contribute to the regression model: Patient's age and gender, type of health insurance, level of education, diagnosis (1 = right, 0 = wrong), and opinion on the questions asked by the doctor's assistant.

^a ^Scale score ranges from 0 to 100, where 0 represents very dissatisfied and 100 represents highly satisfied.

^b ^Scale ranges from 0 to 100: 0 represents not urgent and 100 represents very urgent according to the patient.

* Indicates whether the patient received the type of contact (telephone advice, consultation at the cooperative, or home visit) he or she expected (1 = in accordance with expectation, 0 = not in accordance with expectation)

### Home visits

Almost forty-five percent (402/903) of the patients that received a home visit by a GP from the cooperative returned the questionnaire. About 81% of these patients reported to be satisfied (42.8%) or very satisfied (38.8%) with their contact with the GP cooperative, and 61% thought that the current out-of-hours care was an improvement compared to the former situation. We identified six multi-item scales that represented the patient's opinion on different aspects of out-of-hours primary care, with Cronbach's alpha coefficients between 0.73 and 0.96. Two initial scales have been merged into one scale; these were patient's opinion on the GP's attitude and the treatment by the GP. All other identified scales were the same as the initial scales. Detailed information on the scales and items can be found in table 7.

We found that five variables predicted overall satisfaction, with a variance explained of 51% (see table 5.). Similar to the group of patients who had received telephone advice only, patients who receive a home visit were generally more satisfied when they believed that the GP of the cooperative had made the right diagnosis. When patients were satisfied with the accessibility of the cooperative by phone, the doctor's assistant's attitude on the phone, and the GP's treatment overall satisfaction was higher. In addition, when patients were satisfied about the waiting time until the GP arrives, overall satisfaction increased.

**Table 5:** Regression analysis with overall satisfaction with out-of-hours primary care as dependent variable of patients who received a home visit from a GP from the cooperative. (adjusted R^2 ^= 0.506).

	Unstandardised coefficients		Standardised coefficients		
	
	B	SE	Beta	t	Sig.
(Constant)	-11.650	5.213		-2.235	
Diagnosis ^(1 = right, 0 = wrong)^	11.948	2.461	0.207	4.856	< 0.001
Accessibility by phone ^a^	0.232	0.059	0.198	3.946	< 0.001
Doctor's assistant's attitude ^a^	0.329	0.061	0.282	5.364	< 0.001
GP's treatment ^a^	0.260	0.050	0.233	5.155	< 0.001
Waiting time until GP arrives*, ^a^	0.154	0.030	0.218	5.183	< 0.001

Variables that did not significantly contribute to the regression model: Patient's age and gender, type of health insurance, education level, expectation about type of consultation, urgency of own complaint, and opinion on the questions asked by the doctor's assistant.

^a ^Scale score ranges from 0 to 100, where 0 represents very dissatisfied and 100 represents highly satisfied.

* Single item scale

### Overall satisfaction

The means of the three loci of care, adjusted for age, sex, insurance status, and education level, show that there is no difference between overall satisfaction in the group of patients who visited the GP cooperative (75.1 ± 1.31) and those who received a home visit (72.5 ± 1.37) (Table 6). However, patients who received telephone advice only (66.2 ± 1.30), were significantly less satisfied compared to the other two groups of patients.

**Table 6:** Adjusted means for overall satisfaction.

	Mean	SD	95% CI
Telephone advice	66.2	1.30	63.6 – 68.7
Consultation at GP cooperative	75.1	1.31	72.5 – 77.6
Home visit	72.5	1.37	69.8 – 75.2

^a ^adjusted for age, sex, insurance status, and level of education.

### Non-response

Out of 100 randomly selected patients, who had not returned the questionnaire, we were able to reach 63 by phone. Of these 63 non-respondents 35 (55.6%) were male and 28 (44.4%) were female. Many of them reported that they had forgotten to return the questionnaire (40%). A minority said not to be interested (6.7%) or did not find it needful (6.7%). Most non-respondents (46.7%) gave other reasons like, no time, too difficult, or had lost the questionnaire.

Of these patients, about 71% reported to be satisfied or very satisfied about their contact with the GP cooperative.

## Discussion

The results of this study indicate that patients were generally satisfied about their contact with the GP cooperative. Patients who received telephone advice only, however, were less satisfied compared to those who attended the GP cooperative and those who received a home visit. A small majority believes that current out-of-hours care is an improvement compared to the former situation.

The response rate in our study is not as high as presented previously by others who investigated patient satisfaction with out-of-hours primary care[5,7-9,11]. Reasons for patients not to return the questionnaire in our study were assessed through the non-respondents interview. We found that most patients gave reasons that were not directly related to their contact with the GP cooperative. Therefore, we assume that this reduced response rate may have had little effect on the outcome of our study. In addition, the overall satisfaction in the non-respondents group did not differ much from that of the respondents.

In the process of determining relevant aspects of out-of-hours care to patients, we consulted the province patient organisation and studied discussions on out-of-hours care in newspapers. We have not used patient interviews, although this might have identified other relevant domains of out-of-hours care. However, we think that the current questionnaire captures many relevant domains of out-of-hours care to patients as well as to health professionals.

Based on results of a Danish study [4,5], we expected overall patient satisfaction to be low because our study took place relatively shortly after out-of-hours care had been reorganised. However, we have not assessed patient satisfaction before the reorganisation, and therefore it remains unclear whether satisfaction has changed. Nevertheless, this study showed that more than half of the patients believe that the reorganisation has improved out-of-hours primary care.

We have no reason to believe that the results of this study cannot be generalised to other regions in the Netherlands. Most GP cooperatives in the Netherlands are comparable, with respect to organisation and population size, to those in this study. In addition, the region in our study includes both rural and urban areas. Despite the similarities with out-of-hours primary care in other countries such as Ireland, the UK and Denmark, there are also differences with respect to the way these cooperatives are organised, and therefore care should be taken when generalising these results to other countries.

We identified various factors that are closely related to overall satisfaction. These factors give important insight in aspects of the GP cooperative that really matter in the patient's opinion on out-of-hours care. The patient's opinion on the doctor's assistant's attitude on the phone proved to be the strongest predictor of overall satisfaction with respect to those having received telephone advice and those that received a home visit. Also for those attending the GP cooperative, this factor was a relatively strong predictor; in this group the patient's satisfaction with the GP's treatment was by far the strongest predictor of overall satisfaction. Thus, it appears that the patients' impression of the first contact they have with the cooperative, which is mostly through telephone, strongly influences overall satisfaction.

In accordance with other studies we found that patients who received telephone advice only, are generally less satisfied with the out-of-hours service, compared to those attending the GP cooperative and those receiving a home visit[4,5,8,9,11]. Patient's expectation of care is assumed to be an important factor that influences overall satisfaction[12]. In our study, only 35% of the patients with telephone advice expected that they would receive this type of consultation. In contrast, 85% of the patients that were asked to attend the cooperative or received a home visit found this type of consultation in line with their expectations. This difference in expectation of care may very well explain the difference in overall satisfaction.

It is questionable whether extra information to the public on the process of the telephone triage process will adjust patients' expectations. Similar to what Salisbury et al[8] suggested, we believe that a shift to an out-of-hours care organisation based predominantly on telephone advice may decrease patient overall satisfaction. Therefore, proper information about the telephone procedure at the GP cooperative is desirable[15]. This information can be supplied by the doctor's assistant on the phone, and by written information through folders and posters in GP practices.

## Conclusions

This study has shown that patients are generally satisfied with out-of-hours care, but that patients with telephone advice only are less satisfied than those attending the cooperative or receiving a home visit. The patient's opinion on several aspects of out-of-hours care can predict overall satisfaction, with different predictors regarding the three types of consultations. However, the accessibility by phone and the doctor's assistant's attitude on the phone are always significantly related to overall satisfaction, regardless of the type of consultation. This implies that when trying to improve overall satisfaction one should always focus on at least these two factors. The questionnaire used in this study has potential for use as a standardised instrument for assessing satisfaction with out-of-hours care in The Netherlands for either research or service monitoring.

## Competing interests

The author(s) declare that they have no competing interests.

## Authors' contributions

CU participated in the design of the study, performed the statistical analysis, and drafted this manuscript. AA, SH, PZ, and HC participated in the design of the study, supervised the project, and provided critical edits to this manuscript.

## Pre-publication history

The pre-publication history for this paper can be accessed here:


